# Antiphospholipid Syndrome in a Patient With Autosomal Dominant Polycystic Kidney Disease: The Surface of the Moon

**DOI:** 10.7759/cureus.24014

**Published:** 2022-04-10

**Authors:** John Dayco, Shahzana Shahzad, Hanna Tran, Mohammed Ali, Mahmoud M Musa, Rashid Alhusain, Abdalaziz M Awadelkarim, Navid Mahabadi, Shaheena Raheem, Aris Urbanes

**Affiliations:** 1 Internal Medicine, Wayne State University/Detroit Medical Center, Detroit, USA; 2 Internal Medicine, Wayne State University School of Medicine, Detroit, USA

**Keywords:** deep vein thrombosis (dvt), autosomal-dominant polycystic kidney disease, complicated urinary tract infection, fever of unkown, auto immune

## Abstract

Antiphospholipid syndrome (APS) is a rare coagulopathic disorder diagnosed with a combination of clinical/imaging findings with specific antibody titer elevations over a period of 12 weeks. The following case report will discuss the unusual and challenging hospital course of a patient with extensive autosomal dominant polycystic kidney disease (ADPKD) being treated for a multi-drug resistant urinary tract infection (UTI). The patient later developed multiple deep vein thrombosis (DVT) and was found to have antiphospholipid syndrome. Warfarin, the anticoagulant of choice for antiphospholipid syndrome, has a higher likelihood of intracerebral hemorrhage than direct oral anticoagulants. This is particularly challenging since patients with autosomal dominant polycystic kidney disease have a higher propensity to develop intracranial aneurysms (ICA).

## Introduction

Antiphospholipid syndrome (APS) is a rare disorder in which inflammatory antibodies produced by the body interact with the lining of endothelial cells, causing widespread venous or arterial thrombosis. The exact etiology of APS remains unclear; however, genetic predisposition appears to be a contributor, as well as environmental exposures that may lead to the increased production of specific antibodies. The diagnosis of APS involves an unexplained thrombotic event accompanied by an elevation in either lupus anticoagulant, anticardiolipin, or anti-beta-2-glycoprotein antibodies. The titer elevations must be measured at least 12 weeks apart to exclude transient increases. Although there is no direct correlation between autosomal dominant polycystic kidney disease (ADPKD) and APS, ADPKD has been known to increase the likelihood of urinary tract infections (UTI) [1]. This infectious process likely lowered the patient's thrombotic threshold, uncovering an underlying APS. In this case report, we will discuss an unusual and previously unreported hospital course of a patient with ADPKD who was treated for a multi-drug resistant UTI. The patient remained febrile and was found to have three simultaneous deep vein thrombosis with antibody elevations, leading to a diagnosis of APS. The decision to treat the patient with lifelong warfarin was challenging for our patient and will be discussed further.

## Case presentation

**Figure 1:**
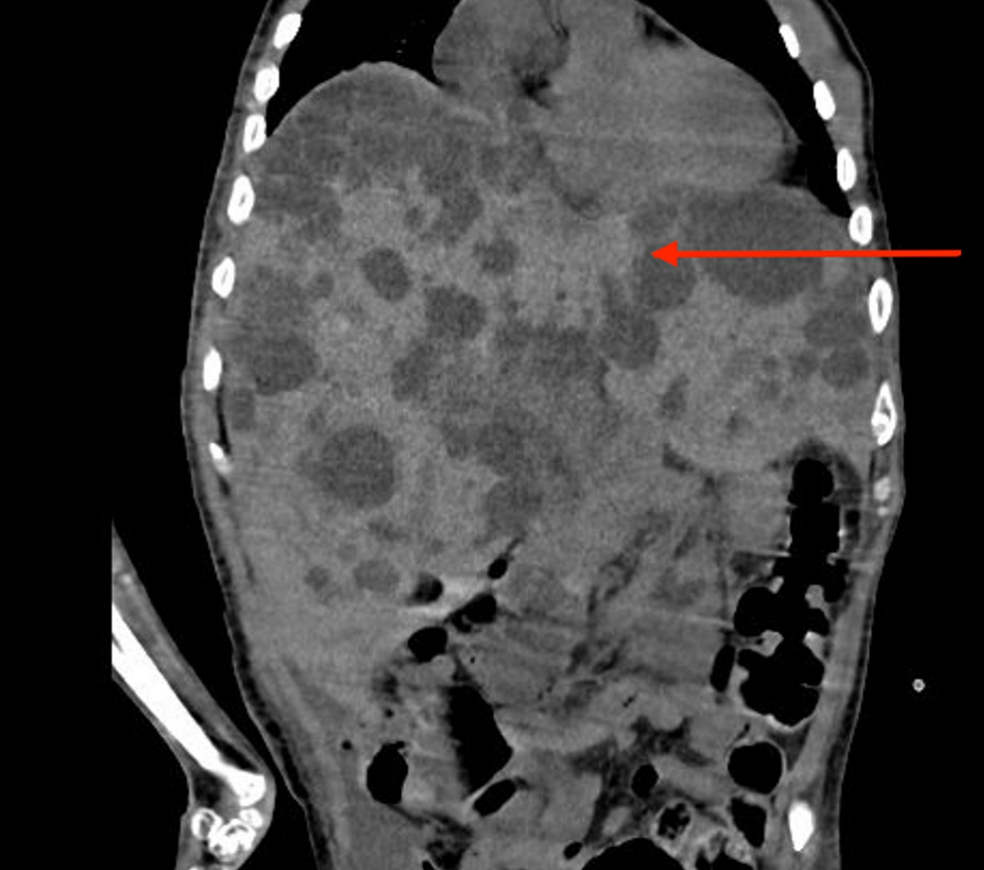
Computed tomography of the abdomen showing innumerable cysts throughout the entire liver (arrow), giving a surface of the moon appearance.

We describe the case of a 51-year-old man with a past medical history significant for ADPKD with extensive liver involvement. The patient was originally admitted for treatment of a complicated urinary tract infection (UTI) with Proteus sp. organisms isolated on urine culture. Despite the completion of a treatment course consisting of intravenous (IV) cefepime, the patient remained febrile. A computed tomography (CT) of the abdomen and pelvis showed innumerable cysts in both the liver and kidneys, which were previously known to the patient. The liver cysts varied from 2 mm to 6 cm and extended throughout the entirety of the liver (Figure 1).

**Figure 2:**
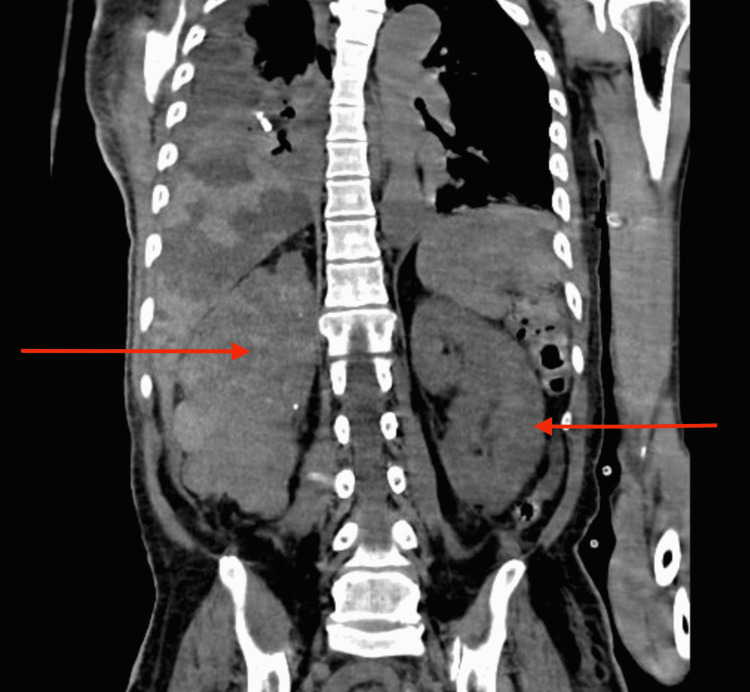
Computed tomography of the pelvis showing extensive bilateral renal cysts (arrow).

The kidney involvement was also extensive, with cysts as large as 3.6 cm observed (Figure 2).

**Figure 3:**
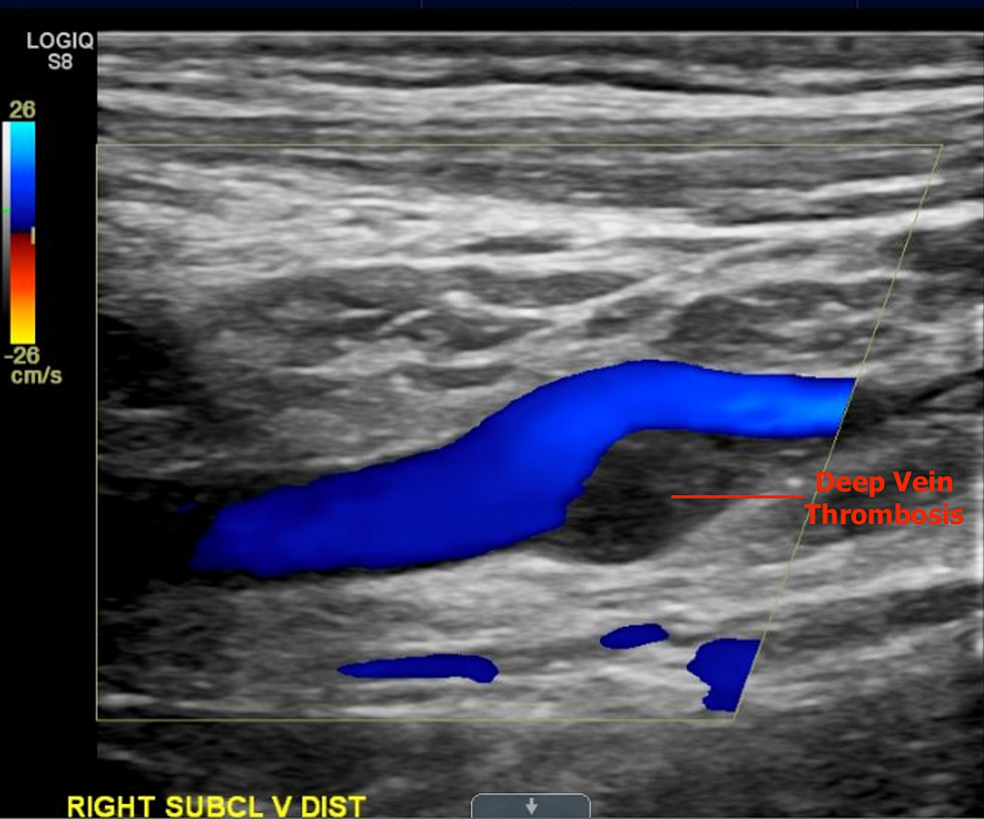
Duplex ultrasound of the upper extremity revealing a deep vein thrombosis in the distal right subclavian vein.

A complete infectious workup, such as blood, urine, and pleuritic fluid cultures, was non-remarkable. A later finding of a mild right lower extremity swelling elicited the decision to conduct a duplex ultrasound scan, which demonstrated a deep vein thrombosis (DVT) in the popliteal vein. Further duplex ultrasound studies revealed extensive DVTs in the right femoral and right subclavian veins (Figure 3).

The sudden onset of DVTs, despite being on subcutaneous prophylactic anticoagulation, prompted a hypercoagulable workup, which was remarkable for an elevation in beta-2-glycoprotein immunoglobulin M (IgM) and anticardiolipin antibodies, suggesting a preliminary diagnosis of APS. The patient was treated with an unfractionated heparin drip of 25,000 units with a target-activated partial thromboplastin time (aPTT) of 60-80 seconds. The patient’s fever resolved, and he was eventually discharged on apixaban, which was the preferred direct oral anti-coagulant (DOAC) for DVTs in our institution. On follow-up 12 weeks later, the patient's serology panel was repeated, which showed persistence in the elevation of beta-2-glycoprotein IgM and anticardiolipin antibody titers, completing the diagnosis of APS. The patient's anticoagulation has now been transitioned to warfarin, with a target INR of 2.0-3.0, the standard of therapy for patients diagnosed with APS [2].

## Discussion

APS is an autoimmune disorder characterized by the persistence of antiphospholipid antibodies (APA) in the presence of arterial thrombosis, venous thrombosis, small vessel thrombosis, and/or pregnancy loss [2]. Antiphospholipid antibodies include anticardiolipin, lupus anticoagulant, or anti-beta-2-glycoprotein-1 antibody. The Sapporo Classification for APS requires the presence of at least one laboratory criteria and one clinical criterion to diagnose APS [3]. Laboratory criteria include the presence of lupus anticoagulant or moderate to high titers of IgG or IgM of anticardiolipin antibody or anti-beta-2-criterion glycoprotein-1 antibody that must be positive in two tests, 12 weeks apart. Clinical criteria include vascular thrombosis and/or pregnancy loss.

The pathophysiology of APS is not entirely clear. It is generally accepted that these antibodies interact with phospholipids, phospholipid-complexes, and phospholipid-binding proteins on endothelial cells. Antibodies against beta-2-glycoprotein-1 seem to be the main pathogenic player, activating endothelial cells, neutrophils, and platelets, which transform the regularly anticoagulant endothelial surface into a procoagulant phenotype [4]. The origin of these autoantibodies is unknown but is most likely a product of interaction between environmental factors, genetic factors, and the presence of inflammation [5]. Some researchers hypothesize APS’s thrombus formation with the "two-hit" model, which explains that a "first hit" injury to the endothelium predisposes the endothelium to a "second hit" injury that promotes thrombus formation [4]. Other targets for APA include complement, tissue plasminogen activator, prothrombin, thrombin, antithrombin, activated protein C, and annexin 2 and annexin V [6].

The patient, with a known history of autosomal dominant polycystic kidney disease, was initially treated for a complicated UTI with Proteus mirabilis isolated from the urine culture. UTIs are quite common for ADPKD patients, with one study estimating that 30-50% of ADPKD patients eventually develop a UTI [1]. Patients with ADPKD have been shown to have an increased number of interstitial inflammatory markers, especially in the renal cysts [7]. The overall inflammatory process associated with the patient's UTI may have played a role in uncovering the patient's previously subclinical APS. If the patient's DVTs were due to the UTI alone, we would expect the antibodies detected on the 12 week follow up to be secondary to the inflammatory response and thus would wane. The persistence of such antibodies is diagnostic of APS. Although inflammation is not a key feature classically associated with APS, multiple studies have demonstrated the role of inflammatory markers in the development of the clinical manifestations of APS [8].

The choice of anticoagulation for our patient is worth discussing. Warfarin is the anticoagulant of choice in APS, and a recent study has demonstrated a threefold reduction in recurrent thrombosis in patients treated with warfarin versus DOACs [9]. However, warfarin has also been associated with an increase in the likelihood of an intracranial hemorrhage compared to DOACs [10]. This is a clinical dilemma for the patient due to the known prevalence of intracranial aneurysms (ICA) in ADPKD [11]. Currently, there are no clear guidelines on the choice of anticoagulation in ADPKD. We initially discharged the patient on apixaban since the diagnosis of APS requires positive serologies 12 weeks apart. In addition, the patient's bed-bound clinical status would have been an additional challenge with regard to the INR monitoring associated with warfarin. Due to the diagnosis of APS in the 12-week follow-up, the patient was transitioned from apixaban to warfarin, per guidelines. The patient would then require close surveillance with magnetic resonance angiography (MRA) for the development of an ICA. If the patient was to develop an ICA, anticoagulation with warfarin would have to be re-evaluated. The degree of MRA surveillance in ADPKD patients on warfarin is also another gap in the literature and deserves to be explored in the future.

## Conclusions

The patient’s unusual hospital course and challenging investigative workup led to a great teaching opportunity. Our ADPKD patient was treated for a complicated UTI and was found to have multiple DVTs, uncovering a diagnosis of APS. The choice of anticoagulation was challenging, as warfarin is preferred for APS, yet it has a higher risk for ICA. This is particularly concerning for ADPKD patients, who have a propensity to develop an ICA. This current gap in the literature deserves to be explored in future studies.
